# Environmental Factors and Personal Characteristics Interact to Yield High Performance in Domains

**DOI:** 10.3389/fpsyg.2019.02804

**Published:** 2019-12-18

**Authors:** Rena Faye Subotnik, Paula Olszewski-Kubilius, Frank C. Worrell

**Affiliations:** ^1^Center for Psychology in Schools and Education, American Psychological Association, Washington, DC, United States; ^2^Center for Talent Development, Northwestern University, Evanston, IL, United States; ^3^Graduate School of Education, University of California, Berkeley, Berkeley, CA, United States

**Keywords:** domain specific talent, psychosocial skills, environmental factors, individual differences, high performance

## Abstract

Outstanding human performance continues to intrigue experts and the public; however, the focus is often on the individual performer or producer with scant attention given to the additive part played by circumstances and contexts. Using general theories of development (e.g., Bronfenbrenner, 1977, 1986, 2005;Sameroff, 2010) and talent development paradigms (e.g., Ziegler, 2005;Dai, 2010;Subotnik et al., 2011), we examined the interaction of environmental and individual factors on trajectories of high performance within and across varied domains. Public and scholarly awareness of the role played by environments places greater responsibility on education and other societal systems to support talents in varied domains, and to promote evidence of talents’ malleability and potential for development.

## Introduction

We begin this article by defining high performance, and the personal and environmental factors that support talent development. Next, we contrast general child development frameworks with those designed to explain talent development. We then provide examples of how personal dimensions work together with environmental contexts to result in outstanding products and performances based on the psychology of high performance in sport, academics, the arts, and professions.

## Definitions of Terms

### Talent Development

Talent development is a process that propels individuals on trajectories from potential to competence to expertise and, sometimes, to eminence (see Olszewski-Kubilius et al., 2016; Worrell et al., 2018). It is driven by opportunities offered within and outside of school and higher education, including exposure to and practice with domain-specific knowledge and mental and social skills. The foundation of talent trajectories includes general and domain-specific abilities and psychosocial skills that are modifiable by education and training, in addition to appropriately timed opportunities. Thus, participants with potential in a domain need to engage in talent development in order to transform their potential into domain-specific abilities and accomplishments. Notably, talent trajectories begin at different developmental periods in different domains, whether based on physiological demands or simply tradition. For example, gymnastics training typically starts in the prepubescent years. During adolescence, expectations for performance in gymnastics are far beyond those for a potential diplomat at that age.

There are several reasons why talent development is sometimes not successful. For example, individual interests do not always align with talents and abilities, resulting in less task commitment than required. Individuals may also avoid opportunities to develop abilities due to fear of failure (Clinkenbeard, 2012). Performance domains such as sport and music co-opt such fears and concerns with intense preparation in psychosocial skills. We argue that along with access to the insider knowledge (e.g., career and educational trajectories, grant opportunities, knowledge of the gatekeepers in the field) and resources (e.g., mentors, scholarships) needed for individuals to achieve their goals, psychosocial skills, like domain-specific abilities, are malleable and can be developed as part of any talent development program.

### High Performance

High performance refers to meeting benchmarks of exceptional accomplishment for each stage in a talent development trajectory, as determined by domain experts and gatekeepers (Subotnik et al., 2019). That is, individuals in the process of developing their talent at one stage need to demonstrate high performance relative to others to move on to the next (e.g., from competency to expertise). By looking at high performance across a range of domains, we can gain insights into how to better understand and facilitate high performance for individuals at all levels of the talent trajectory.

### Environmental Factors

From the moment of conception, individuals are in constant interaction with their environments (e.g., the womb, home, school, society; Bronfenbrenner, 2005). In the context of talent development, environmental factors refer to those that are aimed at propelling the individual along a talent development pathway (Ziegler, 2005). Examples include emotional and financial support from the family, specialized classes, or coaching inside and outside traditional educational contexts, and access to opportunities and experts in the talent domain. Sosniak (1985, p. 417) described this process in the talent development journey of a concert pianist:

Parents began to consider what other activities they could allow their child to engage in without the possibility of harming his or her music making. Parents began making large sacrifices of time and money to get the child to a better teacher, buy a better piano, and travel to competitions.

If an individual who has tremendous potential in mathematics but less potential in other domains is sent to a school for the performing arts rather than a science magnet school, the environment is less likely to support talent development in mathematics. Although typical sibling rivalry does not provide the context for talent development, competing against a sibling who is highly skilled in the same domain – as described by Syed (2010) on his path to becoming a table tennis champion – provides a cogent example of the home environment supporting talent development.

### Personal Factors

Personal factors fall into several categories including general and domain-specific potential and abilities, temperament, personality, psychosocial skills, and mental health. They include wired-in aspects of the individual that are biological in origin. For example, individuals are born with different levels of mathematical cast of mind, musicality, sociability, and tenacity, and these constructs alongside others will interact with each other and the environment and result in differences in accomplishments among individuals. Thus, an individual with superior persistence and high levels of mathematical cast of mind, and an individual with average levels of persistence and superior mathematical cast of mind may both end up as outstandingly creative in mathematics. As Simonton (2005) noted, “most manifestations of giftedness do not depend on the inheritance of just one trait” (p. 271), and “giftedness can develop in contrasting ways for individuals who do not have identical genotypes” (p. 277).

Other personal factors, such as values and beliefs, are learned and acquired as internal standards or principles used to make decisions. These learned characteristics will interact with the inherited ones and can derail or facilitate talent development. For example, all other things being equal, the individual who is more socially adept and appropriately respectful will be more likely to succeed in domains where soliciting finances or patrons to support talent development opportunities are important (Subotnik and Jarvin, 2005; Subotnik et al., 2011). Individual factors can change over time due to influences from within and outside the person, and as noted above, as the person interacts with environmental contexts and chance, producing different talent development outcomes (Subotnik et al., 2011).

## General Child Development Frameworks

We illustrate the cumulative contributions of environmental and personal dimensions to the flourishing of children and youth with brief descriptions of two prominent developmental frameworks – one by Bronfenbrenner (1977, 1986, 2005) and Bronfenbrenner and Morris (2006), and the other by Sameroff (2010). Following these descriptions, we provide examples of selected talent development frameworks that highlight, to different degrees, a balance between environmental and personal contributions to fulfilling potential.

### The Ecological Model of Human Development

In 1977, Bronfenbrenner published a set of propositions based on a series of natural and contrived experiments in which he articulated how environmental forces affect development. Although much of the discussion in Bronfenbrenner’s (1986, 2005) theorizing focuses on the environment, Bronfenbrenner’s central argument can be summarized in this way: development is affected by “the progressive accommodation, throughout the life span, between the growing human organism and the changing environments in which it actually lives and grows” (Bronfenbrenner, 1977, p. 513).

Bronfenbrenner’s (2005) ecology of human development is illustrated as a series of concentric circles indicating different degrees of environmental influence on the individual, ranging from the intimate to distal forces. The first and innermost circle incorporates microsystems. Microsystems include relationships with parents, siblings, and teachers and have the most direct impact on the developing child. The next circle includes mesosystems, which involve interconnections among the microsystems (e.g., home and school, neighborhood and school). Mesosystems, which Bronfenbrenner (1977, p. 515) defined as “a system of microsystems” contribute to development through the various ways microsystems exert influence on other microsystems. For example, the nutrition and fiscal resources in a home can have a profound influence on a child’s ability to learn in the classroom, just as a child’s behavior and academic performance in school can lead to changes in the home (Bronfenbrenner, 1986, 2005).

Several environmental dimensions that are less proximal to the individual can also affect development. Exosystems, encompassing the third concentric circle, are the first of these, and refer to societal and environmental contexts that, although not in direct contact with the individual, nonetheless affect individual development through their influences on the individual’s microsystems and mesosystems. These can include the media, school board policies, the system of government, legal and educational systems, and transportation systems, all of which can have a marked influence on what happens in the school or home and thus affect the developing individual. Beyond the exosystem is the macrosystem – the fourth of the concentric circles – reflecting societal and cultural ideologies and values that determine the customs and practices used in all of the systems already described (e.g., a society’s views on children’s rights). Bronfenbrenner reminded us that the way that society interacts with children is crucial to their chances for optimal development, including whether their talents flourish or languish.

Finally, the chronosystem (Bronfenbrenner, 1986) refers to life transitions (e.g., age of school entry or entry into the workforce) and historical events that can affect development. For example, individuals born in the computer and internet age have a different set of experiences than those born in the 1960s; similarly, the terror attacks by fundamentalists over the past two decades and the ongoing war on terror have changed how Muslim youth are socialized and viewed in many countries around the world.

### Samaroff’s Unified Theory of Development

In a 2010 paper, Samaroff proposed an integrated theory of development incorporating several developmental perspectives. He began with historical trends assigning causation for behavior to nature versus nurture, noting that advances in neuroscience and molecular biology have resulted in nature being preeminent in the first decade of the 2000s, but also pointing out increased recognition over time of the synergy between nature and nurture. After reviewing the concepts of differentiation and integration as non-linear, cyclical forces in developmental and growth models, Sameroff (2010, p. 12) proposed a unified theory based upon an integration of four models “for understanding human growth: a *personal change* one, a *contextual* one, a *regulation* one, and a *representational* one.”

Sameroff’s (2010) theory building provides insights into how we all develop. First, he integrated biological factors such as health and epigenomics with psychological factors such as mental health and social competence as expressed in home, school, community, and the geopolitical world. Second, he addressed change factors such as puberty or new peer groups, incorporating the influence of both traits and developmental stages as well as Bronfenbrenner’s (1977, 1986, 2005) ecological systems, which he called the conceptual model. Third, Sameroff’s integrated perspective subsumes the regulation model, which proposes an interaction between self-regulation and other-regulation, with the former being minimal at birth and increasing over the lifespan, and latter being dominant in infancy and becoming less influential over time. In other words, as individuals mature, self-regulation increases in importance relative to the regulation imposed by or inculcated by others. Finally, the unified model incorporates the representational model, which addresses “encodings of experience” (Sameroff, 2010, p. 16) that are internalizations of the external world, and include cognitive, social, and cultural representations reflected in the “interacting identities, attitudes, beliefs, and attributions of the child, the family, the culture, and the organizational structure of social institutions” (Sameroff, 2010, p. 19).

## Summary

As Bronfenbrenner (1977, 1986, 2005), Bronfenbrenner and Morris (2006), and Sameroff (2010) made clear, development involves both the individual and the environment. Bronfenbrenner (1977) contended that developmental research needed to go beyond the person and the immediate context and investigate not only the larger formal and informal contexts, but also the interconnections among these contexts. Sameroff (2010, p. 20) also emphasized the importance of these interactions:

Neither nature nor nurture will provide ultimate truths and neither can be an end in itself. Instead, each can explain the influences of the other because in the end neither can exist without the other. They mutually constitute each other through their unity and interpenetration of opposites.

We now explore how the contributions of both personal and environmental factors are reflected in talent development frameworks designed to explain outstanding performance.

## Selected Talent Development Frameworks

Several challenges make research and practice in giftedness and talent development especially difficult. The first is that there are no universally recognized definitions of these terms to ensure that study populations consistently represent the concepts under consideration. The goals of talent development are also under debate; for example, which domains are considered valuable enough to warrant public support (Worrell et al., 2019). Nevertheless, several theoretical models have been developed to help organize work conducted in the field, and we provide some examples here. It is noteworthy that all talent development frameworks acknowledge the importance of both the individual and the environment, reflecting basic principles promoted in general developmental frameworks. The talent development models build on these principles to explain the contributions that lead to outstanding performance and creativity in domains of human endeavor.

### Paradigms of Gifted Education

Dai (2010), (see also, Dai and Chen, 2013), described the gifted child paradigm as the traditional view of giftedness. In this view, giftedness is operationalized with general intelligence or IQ (nature) and the goal of gifted education is to provide appropriate educational opportunities (nurture) to facilitate development of these intelligent children’s potential. Dai and Chen contrasted the gifted child concept with the talent development paradigm (described subsequently), and finally a differentiation paradigm. The differentiation paradigm in gifted education is focused on subjects taught in school. Labeled Advanced Academics (McBee et al., 2012; Peters et al., 2014), this approach addresses differentiation within the school context in the form of curricular and instructional adaptations (Robinson and Robinson, 1982). Rather than using general intelligence as a marker, this paradigm suggests looking at performance in mathematics, or language arts, or other academic content, identifying the students’ specific academic level, and adapting the curriculum to meet student needs. Except for its narrower focus on the classroom and school level, advanced academics is compatible with the talent development paradigm, which is exemplified by two models described in more detail below.

### Actiotope Model of Giftedness

Actiotope (Ziegler, 2005) is a dynamic model that focuses on interactions between potentially talented people and their environment, animated by adaptation and regulation. As individuals with appropriate abilities and drive work to meet their goals, they adjust in response to successive learning challenges. Those who aspire to excel in a domain acquire both educational and learning capital. Learning capital resources are inherent to the person. They may include physical and health capacities, specific abilities, goals and aspirations, and self-regulation. Educational capital is derived from the environment and capitalized upon by the individual. It includes, for example, systems of instruction, resources of time and money, cultural values and opportunities, and social systems that can enhance or impede progress. Actiotope reminds us that life changes are the constant and that we need to focus our attention on the fluid dynamic between personal and environmental as well as the benchmarks of talent development.

### Megamodel

The megamodel (Subotnik et al., 2011, 2018a,b) is premised on principles of talent development derived from a comprehensive review of the psychological science literature in the academic, sport, and arts domains: (1) abilities, especially domain-specific abilities, are malleable and need to be developed to fulfill potential; (2) talent trajectories vary by domain in when they begin, peak, and end; (3) talent development requires the provision of opportunities both inside and outside of school and into careers; (4) these opportunities must be taken up by the talented individual; and (5) over time, taking opportunities and maximizing one’s talent are increasingly based on the development and acquisition of psychosocial skills. Principles 1 and 5 are prime examples of the *interaction* of the personal (nature) and environmental (nurture) dimensions. Principle 2, an environmental dimension, incorporates biological factors with the culture of talent development trajectories that have emerged based on tradition. Principle 3, another environmental factor, points toward the different ecological contexts, and Principle 4 describes the responsibility of the individual to engage in talent development, harkening back to Sameroff’s (2010) discussion of self-regulation.

## How Environmental and Individual Factors Work Together Cumulatively to Influence Talent Development and High Performance

Drawing from Bronfenbrenner (2005) and Bronfenbrenner and Morris (2006), factors external to a talented individual can influence whether the expression of talent is valued and developed or denied. Early experiences with artists, athletes, or scientists result in advanced familiarity with doing well in those domains (Almarode et al., 2017; Olszewski-Kubilius et al., 2017). Schools can either reinforce the value placed by families on sport, academics, or music – or not. Finally, culture (familial, neighborhood, and national) and socioeconomic status profoundly affect how young people choose to or are able to expend their time and efforts (Olszewski-Kubilius et al., in press). Musical instruments and lessons are expensive, as are special sport accoutrements such as golf clubs and golf course memberships. High-quality teachers are often inequitably distributed, with more inexperienced instructors assigned to high poverty schools. Co-curricular opportunities are fewer and farther between in communities without a tax base to support museums, orchestras, ball fields, or innovative industries.

Families with multiple generations of financial stability and accumulated cultural and financial capital are much more likely to support their children’s pursuit of a creative career that requires a longer and more substantial commitment as well as dubious financial payoff for the individual and the family. Thus, children from families that are experiencing instability may be at risk for failing to develop their creative talents. Families that are striving for upward mobility might exert considerable pressure on their children to follow educational paths toward conventional and lucrative careers rather than what are considered “iffy” creative professions in the arts or lower paying jobs in the helping professions. Families that are marginalized in a society as a result of race, ethnicity, family structure, or SES may also eschew traditional educational paths and professions based on the belief that the financial and status rewards typically associated with those paths will not be the same for their children. These families may push their children toward professions such as sport and entertainment that they perceive are more open to and accepting of their group and have a quicker payoff (Olszewski-Kubilius et al., 2017). These are ways in which family status, and specifically parental values, influence opportunities for the recognition and development of children’s talents and abilities.

Gender and birth order, particularly, but not exclusively in families that are struggling financially, can influence the distribution of family resources, including money and parental time and attention, thereby influencing opportunities such as higher education or participation in supplemental programs as well as pressure toward particular career choices. First-born children and males may have an advantage in these families. A physical or learning disability can result in parents protecting a child to the extent that talent is unnoticed and underdeveloped or, alternatively, spur parents to focus a great deal of time, attention, and resources toward ensuring the child’s opportunities and talent development are not limited nor compromised. Immigrant families as well as parents who did not themselves experience success within school may feel less equipped to advocate for their child in the current educational system. Alternatively, parents who themselves were less successful in school or perceived that they received an inadequate education may be relentless advocates for better opportunities for their children (Olszewski-Kubilius et al., 2018).

Family discord and dysfunction can deter talent development, sapping energy from parents’ ability to cultivate a home environment that supports achievement and from children’s ability to engage in learning in school. Alternatively, a less than harmonious family environment may produce psychologically independent children who are motivated to prove themselves, have remarkable coping skills, and are extremely resilient in the face of environmental stress and obstacles – all of which will serve them well on the path to talent development. An individual may choose to heal a childhood trauma in a way that maximizes talent (e.g., becoming a doctor after experiencing gang violence) or in a manner that negatively exploits it (e.g., leading a gang). What makes a difference in the paths that individuals take given their experiences and family backgrounds?

One contributing and intervening factor is the influence that parents and other significant others have on their children’s beliefs and values, and ultimately their actions and decisions, through the interpretations they provide for significant events that affect the family and child – both within the immediate context and from the broader society. Messages that emphasize positive coping, optimism, hope, resiliency, and self-efficacy can greatly influence students’ commitment and persistence to engaging in arduous talent development trajectories within domains. Research indicates that students who are more hopeful report lower levels of perceived stress and higher levels of belonging, self-esteem, educational expectations, perceived life chances, and achievement than their less hopeful peers (Dixson et al., 2017). Supports outside the immediate family, such as caring and attentive teachers, coaches, extended family, and mentors, and outside of school or community programs can compensate for what may be lacking in the immediate family environment and facilitate talent identification and development.

Kiewra (2019) studied adolescents who had excelled in diverse fields such as baton twirling, skating, swimming, equestrian arts, and chess with a particular focus on the contribution of families to their children’s accomplishments. He identified a number of ways in which families supported their children, including accessing opportunities in their talent area, finding teachers and coaches, managing their children’s schedules so that they can participate in competitions and lessons, and providing both emotional and financial support. In his study, Kiewra reported on the great lengths parents went to to support their children – taking loans to pay for lessons, moving to be near to better coaches and teachers, and even creating opportunities (e.g., chess clubs) where none existed – findings also supported by the early work of Bloom (1985) across diverse talent areas.

The family is just one context in which the developing individual participates, although a primary and extremely influential one. As described by the Actiotope Model (Ziegler, 2005), development occurs *in situ* and results from a complex interaction of person variables and environmental influences. Additionally, the influence is bi-directional, with child characteristics eliciting responses and actions from parents and others and parental actions influencing the development of beliefs, attitudes, values, and personality characteristics of children. Kiewra (2019) noted that although high-achieving adolescents are perceived to have pushy, over-involved parents, in the talented adolescents he studied parental support was led by the intense interest and passion of the child for the talent domain, or what Winner (1996) termed “a rage to master.” Children led the way and parents followed with support and resources.

MacNamara et al. (2010a,b) studied the role of psychosocial skills in facilitating pathways toward elite performance in several areas of sport (team and individual) and in music. They asked elite performers to map their trajectory over time and found that rather than a linear path, the participants experienced wave-like patterns of highs and lows across all domains. Although there was considerable individual variation even within fields, classical musicians encountered ups and downs earlier in their trajectories than did rugby, hockey, or track and field athletes. The authors speculated that early success in some fields, such as track and field, may be related more to natural talent, greater physical maturity, and an appropriate physique. In contrast, other fields require considerable investment in the acquisition of technical and tactical skills (e.g., hockey, classical music, gymnastics, ballet, figure skating) before one can perform well. Thus, personal characteristics may have more influence on initial success in some domains than others, but eventually psychosocial skills become critical in all domains.

Movement to elite levels of performance in all fields requires motivation, deliberate practice or consistent study, and perseverance, but some fields such as music may demand this earlier than others and individuals who possess these skills and personal characteristics will be at an advantage (MacNamara et al., 2010a,b). Some individuals will be deterred by setbacks and perceived failures, such as not being chosen for a team, losing a game or match, sustaining an injury, or losing a competition, and these individuals stop making progress. Alternatively, other individuals will be spurred on by these same experiences to focus on improving their skills, strengthening their commitment, and investing even greater amounts of time and energy. Whether the performer is demotivated or inspired depends on the athlete’s or musician’s beliefs about their ability, their confidence, and their coping and psychosocial skills. MacNamara et al. (2010b) noted, “The extent to which these micro stages and transitions were experienced as facilitators or debilitators varied considerably and was dependent on how they were interpreted by the individual” (p. 87). Thus, staying on a trajectory toward elite performance is very much dependent upon the interaction between the context (*environmental* aspects of the performance domain) and the characteristics of the *individual* (e.g., age, cognitive maturity, personality).

For all domains of talent, a key transition takes place when individuals take charge of their own talent development and are less reliant on coaches, trainers, and teachers (MacNamara et al., 2010a). This transition involves setting performance and practice goals and engaging in deliberate practice independently. In order to improve their performance, athletes might change coaches and musicians might change teachers. They may also employ extrinsic rewards to help them engage in long, strenuous periods of practice. Several athletes in the MacNamara et al. study noted that they felt they had less aptitude than some others in their sport but had greater drive and willingness to work hard to improve. They witnessed teammates who had enormous talent and potential but who did not transition to elite levels of performance because they did not invest fully in training and practice or come back from failures and defeats. What differentiated the successful musicians and athletes from less successful ones was what the authors termed psychological characteristics of developing excellence, or “PCDEs (MacNamara et al., 2010a).” PCDEs include motivation to succeed, determination, perseverance, pursuit of excellence as a priority, having a vision of what it takes to develop further, goal setting, focus and distraction control, the belief that one can excel, and pressure management.

MacNamara et al. (2010a) suggested that deliberately teaching PCDEs will enable many more individuals with talent to reach higher levels of performance. How elite-level athletes acquire PCDEs is an open and important question for researchers. Many of the elite performers in this study noted that initially they had coaches, parents, and teachers who set practice times and goals for them – that is, these individuals ensured that they engaged in practice and provided emotional support especially in times of struggle and uncertainty (other-regulation). The elite performers gradually assumed the management of their own talent development (self-regulation), perhaps influenced by the modeling or direct teaching of their coaches and instructors and driven by their own desire to improve their performances. Clearly, personal characteristics of the talented individual interact with environmental opportunities and supports to create synergies that help or hinder talent development.

### Creative Production

An important goal for the talented individual who seeks to contribute to a field is to generate a creative performance, product, or idea, and make sure colleagues and gatekeepers know about it. No matter the domain, the farther along the trajectory toward eminence, the more likely abilities and acquired techniques, experience, and knowledge are taken for granted. In comparison, psychosocial skills and insider knowledge become increasingly important. Being creative requires courage, self-confidence, concentration, preparing for setbacks, and knowing how the “game” is played. We provide here some examples from the professions and the arts.

Becoming a physician is a long and laborious process. Novices pass through multiple hoops of coursework that require rote memorization and practice. Other than newly informed requirements for reasonable bedside manner and understanding behavior associated with patient compliance, progress is gauged using standardized tests of knowledge acquisition. Success with these early challenges opens doors to a range of specialties, and it is here that excellence is determined by matching the demands of the work and the personal characteristics and values of the individual. Emergency room and trauma surgeons need to remain calm even under the most difficult conditions and provide leadership to teams of medical personnel. Pathologists, anesthesiologists, and radiologists have fewer interactions with patients, yet sometimes are called upon to make decisions or draw conclusions in high-stress situations such as the operating room or courtroom. In medicine and in fields such as software engineering, the most creative outcomes are derived from inspiring colleagues and mentees with a “churn of ideas,” including methods for developing new techniques and methods for working productively with healthcare colleagues (McWilliams et al., 2019).

The talent development process of elite classical musicians is, like medicine, relatively traditional, and varies little by country or region of the world. The most significant decision in a career is the match between student and teacher. This match process begins with auditions at a music conservatory where admission is based on whether one of the instructors chooses to take on the candidate. Most of the instruction is conducted one on one. Each teacher conveys the skills and knowledge accumulated from a lineage of her own teachers, sometimes going back decades or even centuries. Students must decide whether or when to break from their teachers’ distinct style and forge their own identity. Young performers must also make judicious decisions with regard to repertoire, managers, and whether to aspire to a solo or orchestral career (Jarvin and Subotnik, 2010).

The culinary arts have changed dramatically from a craft left to servants to one where chefs are celebrated for productions that are both edible and aesthetically pleasing (Aron et al., 2019). During the initial stages of development in the culinary arts, the focus is on the acquisition of techniques for working with various stations in the kitchen, being able to work quickly and respond to changes or problems with alacrity. Over time, a developing chef will work with more “precious” products and conduct more “noble” tasks. To achieve eminence, a chef will need to establish a signature dish or approach and learn to successfully manage the kitchen as well as charm reviewers and clients in the dining room. Again, at higher stages of talent development, personality and psychosocial skills become critical ingredients to success.

## Suggestions for Future Research in High-Performance Psychology

There is a great deal of variation in how well domains are researched (Worrell et al., 2019). Domains with high economic stakes like major league sports, as well as music and business have richer bases of scholarship. Required abilities, benchmarks of success, advantageous psychosocial skills, and insider knowledge are relatively well documented. Within-domain comparisons remain exciting places for discussion and investigation. For example, how do early versus later specializations in sport (gymnastics vs. team sports) differ in terms of abilities, benchmarks, psychosocial skills, and insider knowledge?

Other domains with longer histories of empirical research include drawing and mathematics. Although research in drawing has not had lots of fiscal support, mathematics research has a long history of targeted federal funding as well support from the financial industry. In both of these domains, scholars have been able to identify precursors to future achievement. What is less obvious, however, is what leads to *creative* production beyond deep understanding and commitment.

Domains with robust bodies of research are ripe for policy development, more specifically, policies that can help institutionalize and promulgate talent development in those domains in schools, school systems, or communities. These policies might then serve as models for other domains as they become more evidence based. Many domains of talent are under researched. This may be due to (1) little to no funding associated with study in this area such as creative writing by children, or (2) because the domain is culturally situated such as circus arts or drum corps, or (3) because there are just so many talent domains that a society can support and recognize. Research questions in these domains are wide open, and we hope that young scholars will pursue work on abilities, benchmarks of performance, psychosocial skills, and insider knowledge with gatekeepers and eminent practitioners in each field.

## Conclusion

Just as general development proceeds *via* the interaction of nature and nurture or the individual and the environment (Bronfenbrenner, 2005; Sameroff, 2010), talent development leading to outstanding performance and sometimes eminence is also dependent on interactions between individuals and the environment (Ziegler, 2005; Subotnik et al., 2018a,b). Although serendipity plays a role, it is also clear that talent development cannot be left to chance alone (Sosniak and Gabelko, 2008; Subotnik et al., 2011). In addition to potential particular to a domain, talent development also requires specific types of environments (e.g., knowledgeable teachers, coaches) and specific types of responses to environmental pressures (e.g., persistence, engaging in deliberate practice). Without an accumulation of all of these interacting factors, talent development is not likely to occur, and potential will remain an unfulfilled promise.

## Author Contributions

The three authors collaborate on a number of projects and the work is conducted seamlessly. The topic of the submission is derived from work initially funded by the association for psychological science in 2009.

### Conflict of Interest

The authors declare that the research was conducted in the absence of any commercial or financial relationships that could be construed as a potential conflict of interest.
